# Sparse and Dispersion-Based Matching Pursuit for Minimizing the Dispersion Effect Occurring When Using Guided Wave for Pipe Inspection

**DOI:** 10.3390/ma10060622

**Published:** 2017-06-06

**Authors:** Javad Rostami, Peter W. T. Tse, Zhou Fang

**Affiliations:** Smart Engineering Asset Management Laboratory, Department of Systems Engineering and Engineering Management, City University of Hong Kong, Tat Chee Avenue, Kowloon, Hong Kong, China; javad.rostami@ymail.com (J.R.); zhoufang-c@my.cityu.edu.hk (Z.F.)

**Keywords:** Ultrasonic Guided Waves, signal processing, NDT, pipe inspection

## Abstract

Ultrasonic guided wave is an effective tool for structural health monitoring of structures for detecting defects. In practice, guided wave signals are dispersive and contain multiple modes and noise. In the presence of overlapped wave-packets/modes and noise together with dispersion, extracting meaningful information from these signals is a challenging task. Handling such challenge requires an advanced signal processing tool. The aim of this study is to develop an effective and robust signal processing tool to deal with the complexity of guided wave signals for non-destructive testing (NDT) purpose. To achieve this goal, Sparse Representation with Dispersion Based Matching Pursuit (SDMP) is proposed. Addressing the three abovementioned facts that complicate signal interpretation, SDMP separates overlapped modes and demonstrates good performance against noise with maximum sparsity. With the dispersion taken into account, an overc-omplete and redundant dictionary of basic atoms based on a narrowband excitation signal is designed. As Finite Element Method (FEM) was used to predict the form of wave packets propagating along structures, these atoms have the maximum resemblance with real guided wave signals. SDMP operates in two stages. In the first stage, similar to Matching Pursuit (MP), the approximation improves by adding, a single atom to the solution set at each iteration. However, atom selection criterion of SDMP utilizes the time localization of guided wave reflections that makes a portion of overlapped wave-packets to be composed mainly of a single echo. In the second stage of the algorithm, the selected atoms that have frequency inconsistency with the excitation signal are discarded. This increases the sparsity of the final representation. Meanwhile, leading to accurate approximation, as discarded atoms are not representing guided wave reflections, it simplifies extracting physical meanings for defect detection purpose. To verify the effectiveness of SDMP for damage detection results from numerical simulations and experiments on steel pipes are presented.

## 1. Introduction

Pipes carrying gas, water or chemicals are important infrastructures in big cities. In order to avoid any trouble being caused by any rupture, their integrity must be checked on a regular basis. Ultrasonic guided wave is an effective nondestructive testing (NDT) tool for checking the normality of these structures and detecting probable defects caused by corrosion or other environmental elements [1,2,3,4,5]. Dispersion, multiple modes, and noise are common problems associated with using guided waves [6,7,8,9,10]. As guided wave propagates, dispersion makes its energy spread out in space and time. It is illustrated as an increase in received the signal duration in comparison with that of the input signals. In practice, dispersion deteriorates signal resolution and complicates its interpretation. Using signals with limited narrow bandwidth signals can reduce dispersion effect. This is because of the concentration of the input energy over a limited frequency band so that any changes in the velocity of the desired guided wave mode are small. This ultimately infers operating at points on the dispersion curves in which the group velocity is almost stationary with respect to frequency. Nevertheless, the dispersion cannot be eliminated. It is worth noting that as dispersion occurs to the guided waves as they propagate in the structure, it cannot be over-come by sensor design either [11]. Meanwhile, because of its boundary condition, guided waves consist of multiple spreading and overlapping modes. In practice, it is very difficult to excite a pure mode; plus, mode conversion when guided wave reaches a defect causes overlapping different wave packets and worsens the signal complexity. Furthermore, as guided wave signals are subjected to contamination from a diverse range of sources, the problem becomes even more challenging. Such sources may randomly produce environmental noise and unwanted modes as a result of mode conversion and reverberation.

In order to over-come the abovementioned difficulties, two different approaches could be taken into account: sensor design and signal processing. Guided waves can be generated and received by a variety of methods. A considerable amount of efforts in past two decades were focused on developing a transduction system for pure mode excitation that receives signals with maximum signal to noise ratio (SNR). As the performance of ultrasonic contact probes due to certain technical factors associated with using couplant is not satisfactory, non-contacts methods have been developed for more efficient transduction [12,13,14,15,16,17]. For example, several scholars have reported the development of magnetostrictive sensors that are suitable for narrowband excitation and are cost-effective, durable and flexible [18,19,20,21]. Sensor design can be used to suppress unwanted modes but as mentioned earlier, it cannot get rid of the dispersion and its subsequent signal complexity. In a different approach, guided wave signal processing can play a key role in the accurate evaluation of structures. In order to over-come the challenge of interpretation of complex guided wave signals, making use of an advanced signal processing tool is inevitable. While Fourier Transform demonstrates the frequency components of signals, because of non-stationary nature of guided wave signal, it fails to explain inherent complication caused by mode mixing. Time-frequency representation (TFR) analysis can be useful for investigation of guided wave signals. There has been a considerable amount of work on improving TFRs suitable for guided wave signal processing with taking the dispersive nature of guided waves into account [22,23,24,25,26]. Nonetheless, if a signal contains overlapped modes within the same frequency band as the excitation signal, utilizing TFRs may not be very helpful. Noting such constraint, Matching Pursuit (MP) could be a good candidate for wave separation. MP is a greedy algorithm that finds the best match for a signal in from an over-complete and redundant dictionary. This method has already been reported for guided wave inspection and defect characterization [27,28,29,30,31,32]. However, the majority of the efforts in MP for guided wave inspection have neglected the dispersive nature of guided waves. Some scholars have reported utilizing Chirplet Matching Pursuit and tried to estimate the dispersion by chirp functions [31,33,34]. Despite being effective, these methods suffer from mathematical complications. Noting that prediction of dispersion has no analytical solution [9], only numerical methods can be used to speculate this guided wave feature. It has been shown that FEM can effectively model such problems. In the past, the computational cost of simulating guided wave propagation was prohibitively high. Advances in computer power have made FEM an excellent candidate to simulate guided wave propagation. In commercially available FEM software such simulations do not require much time [35,36].

Addressing the three abovementioned problems in guided wave signal interpretation, we design a dictionary that is based on actual waves obtained from the finite element simulations. Moreover, a two-stage algorithm is designed based on such dictionary to represent guided wave signals with the maximum sparsity. In the first stage, a signal is approximated and in the second stage, sparsity is further increased based on the frequency components of the excitation signal. Therefore, unwanted components of guided wave signals are eliminated and at the end, a very clean signal with meaningfully decomposed components remains for further analysis.

In this paper, after briefly reviewing the theory of Mating Pursuit, the idea of designing a dictionary with consideration of dispersion effect is explained. Finite element approach that is taken into account is described for designing a dictionary for pipe inspection. The main idea of designing the new Sparse and Dispersion-based Matching Pursuit (SDMP) for minimizing the dispersion effect of guided waves due to the increasing length of wave propagation is explained in a later section and at the end; the proposed idea was verified with FEM results and experiments.

## 2. Matching Pursuit

Matching Pursuit (MP) is a greedy algorithm that iteratively approximates a signal *x*(*t*) by selecting the atom *a*(*t*) from a redundant dictionary *D* that best matches the signal [37]. If the dictionary is designed based on well-known characteristics of the system that generate a signal, only a few atoms are required to represent the signal. The dictionary is a matrix whose columns are well-known elementary signals with the same sampling frequency as the signal *x*(*t*). The best match in the iteration is the one that produces the maximum inner product with the signal. From a mathematical point of view, MP represents a signal as a linear combination of some atoms *y_n_*(*t*) plus a residue *r_n_*(*t*) after *n* iteration:

(1) $$\begin{array}{l} x\left(t\right)=y_{n}\left(t\right)+r_{n}\left(t\right)=\sum_{i=1}^{n}c_{i}a_{i}\left(t\right)+r_{n}\left(t\right)\\ =\sum_{i=1}^{n}\langle x\left(t\right),a_{i}\left(t\right)\rangle a_{i}\left(t\right)+r_{n}\left(t\right),\\ \left\|a_{i}\left(t\right)\right\|=1 \end{array}$$

where *c_i_* is the corresponding coefficient of atom *a_i_*(*t*) determining its amplitude and is a real number. The iteration in MP continues until the second-order norm of the *r*(*t*) for optimum approximation becomes minimum [38]:

(2) $${\left\|r_{n}\left(t\right)\right\|}_{2}={\left\|x\left(t\right)-y_{n}\left(t\right)\right\|}_{2}<\varepsilon$$

in which *ε* is a pre-defined value depending on the noise level in the signal (usually unknown and must be set by trial and error) [28]. Assuming the signal has been approximated by *n* − 1 ≥ 0 atoms, for further approximation by *n* atoms the MP algorithm in consecutive steps is as follows:

(1) Calculate the inner product between the residue *r_n−_*_1_(*t*) and all atoms in *D* excluding the ones that were already selected in the previous iterations.
(2) Select an atom *a* that produces the maximum inner product with *r_n−_*_1_(*t*):
  (3) $$\left|\langle r_{n-1},a_{n}\rangle \right|\geq \rho \sup\left|\langle r_{n-1},a\rangle \right|,a\in D$$
  where 0 < *ρ* ≤ 1 is some number independent of *n*.
(3) Compute the new residue:
  (4) $$r_{n}\left(t\right)=r_{n-1}\left(t\right)-\langle r_{n-1}\left(t\right),a_{n}\left(t\right)\rangle a_{n}\left(t\right)$$
(4) The new approximation of the signal *x*(*t*) with n atoms can be represented as:
  (5) $$x\left(t\right)=\sum_{i=1}^{n}\langle x\left(t\right),a_{i}\left(t\right)\rangle a_{i}\left(t\right)+r_{n}\left(t\right)$$

## 3. Dictionary Design for Guided Wave Inspection

**Figure 1:**
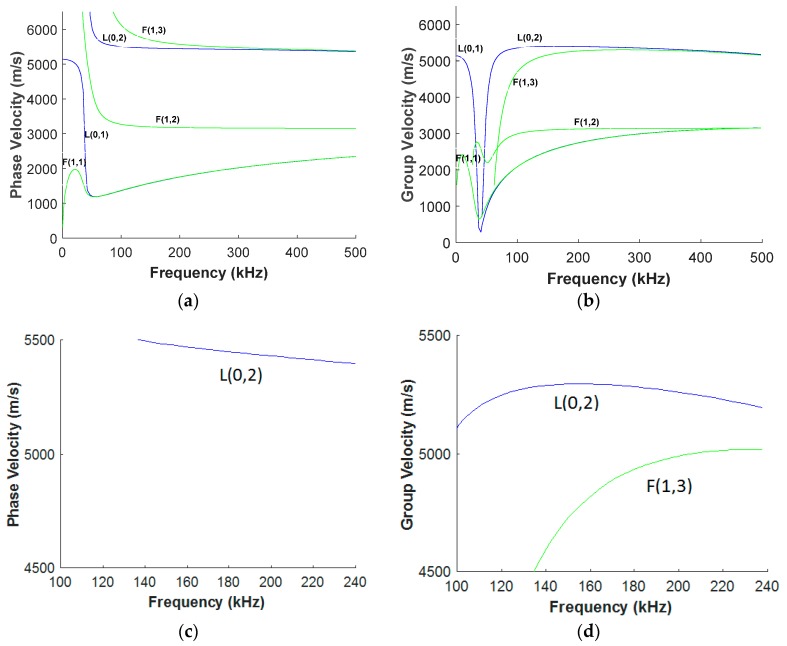
(**a**) Phase; and (**b**) group velocity dispersion curves for a steel pipe with a diameter of 33 mm and 4 mm in wall thickness. (**c**) Phase; and (**d**) group velocity dispersion curves for the same pipe within the least dispersive frequency region.

For a successful decomposition and approximation of a guided wave signal with matching pursuit, designing an appropriate dictionary is of vital importance. This design must be based on prior information about the propagation of guided waves in a structure. Meanwhile, over-completeness and redundancy of the dictionary must be taken into account. Being overcomplete implies that the number of atoms in a dictionary exceeds the length of a signal to be approximated. Redundancy denotes that the atoms do not necessarily satisfy the orthogonality. Such properties maximize the matching of waveforms between the atoms and the signal. If the dictionary is properly designed only a few atoms are required to represent the signal. One of the major characteristics of guided waves that should be taken into account in dictionary design is dispersion. Dispersion means that the velocity of guided waves is a function of its frequency. The dispersion relation for every structure is represented by dispersion curves (Figure 1). For guided wave inspection, the least dispersive region is preferred. Nevertheless, as can be seen in Figure 1, within the least dispersive region, a variation of the velocity is not negligible.

**Figure 2:**
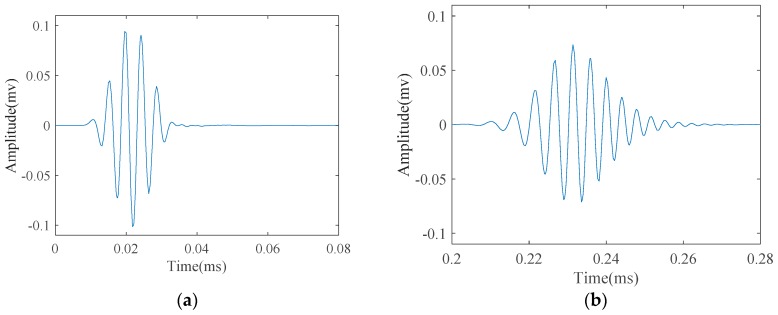
L(0,2) mode as emitted a five-cycle tone-burst signal in a normal pipe after propagating a distance of: (**a**) 50 mm; and (**b**) 1050 mm along the pipe axial length.

Being dispersive, the energy of a wave packet propagates at different speed depending on its frequency. As the wave packet propagates along the structure, it spreads in time. This is illustrated in Figure 2, which demonstrates the propagation of L(0,2) mode in a steel pipe with a diameter of 33 mm and 4 mm in wall thickness. The wave-packet after propagating 50 mm looks almost the same as excitation signal, which was a five-cycle tone-burst signal with a center frequency of 220 KHz (Figure 2a). Nonetheless, after traveling 1050 mm, it is distorted from the original shape and spreads in time by increasing the duration of the wave-packet, which consequently decreases the signal amplitude (Figure 2b). Such reduction in amplitude of a dispersive wave-packet causes the reduction of sensitivity for defect detection purpose.

**Figure 3:**
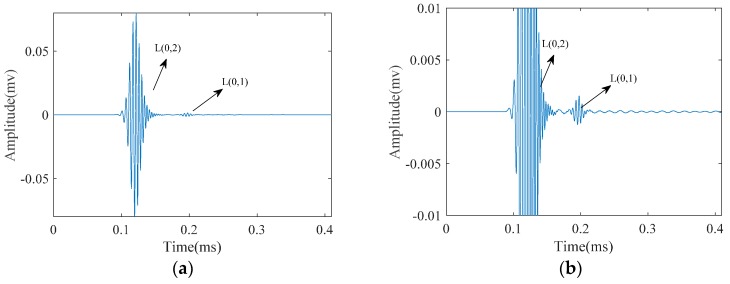
Longitudinal guided wave modes emitted at a 220 kHz center frequency as a five-cycle tone-burst signal into: (**a**) a pipe; and (**b**) the zoomed wave signal.

Meanwhile, when it comes to practical application of guided waves, it is always very difficult to have a pure mode excitation. If longitudinal guided waves are selected to test a pipe, L(0,1) and L(0,2) modes will be generated at the same time. Though L(0,2) mode is the dominant mode with the maximum velocity, L(0,1) mode is separated from the main wave package after traveling some distance (Figure 3).

Considering these two main features of guided waves, we propose designing a dictionary based on real guided wave data. However, as it is extremely difficult and time-consuming to conduct such extensive experiment for collecting the essential data for the dictionary, we propose taking finite element method (FEM) into consideration. Guided wave propagation can be simulated in a desired geometry and material with pre-defined characteristics including waveform, central frequency and excitation type and material characteristics.

Dispersion of guided waves in designing a dictionary for matching pursuit was addressed by Hong et al. [34]. They used chirp functions as dictionary atoms to simulate dispersion phenomenon. Utilizing chirp functions for matching pursuit was further computationally improved by Raghavan and Cesnik [33]. In this paper, we take a different approach for construction of the dictionary by considering dispersion and existence of unwanted modes. In doing so, instead of designing a chirp function and defining multiple parameters and optimize them in order to obtain the best matching atoms, real guided wave signals are used as dictionary atoms. The main idea is to collect the guided wave signals in equally spaced steps on a structure so that at every location from the excitation point, the wave-packet shape is identified. Nonetheless, it is cumbersome and time-consuming to obtain such database by experiments.

Apart from analytical approaches to resolving guided wave propagation problems [9,39], numerical methods can accurately predict guided wave behavior in structures [40,41,42,43,44]. On the topic of numerical simulation of guided waves, there have been a lot of works on using finite element methods (FEM) for calculating dispersion and waveform prediction [36,45,46,47]. Using a commercially available finite element code [36] the experiments required for the dictionary database can be simulated. If on a structure such as a pipe the number of the selected points for data collection along the pipe is large enough, the overcomplete redundant dictionary can be achieved. As our focus in this paper is on guided wave inspection for pipes, simulation and subsequently data collection on a steel pipe is explained. For simulation of a steel pipe whose properties are tabulated in Table 1, the 2D axisymmetric model was used to save computational time. The simulated pipe has a 33 mm diameter and 4 mm wall thickness.

For the narrowband excitation which is the case for the most guided wave applications for pipeline inspection, a five-cycle tone burst signal modulated with Hamming window (*S*(*t*)*_excitation_*) was chosen:

(6) $$S{\left(t\right)}_{excitation}=\sin\left(2\pi ft+\theta \right)\left(0.08+0.46\left(1-\cos\left(\frac{2\pi ft}{5}\right)\right)\right)$$

**Figure 4:**
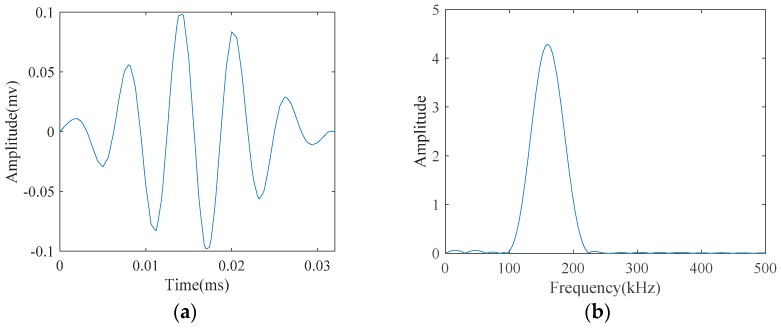
The desired five-cycle tone-burst emitted at 160 kHz as the center frequency in: (**a**) its temporal waveform; and (**b**) its spectrum.

In the above mentioned equation *t*, *f* and *θ* are the time, central frequency and phase, respectively. Figure 4 demonstrates this excitation signal together with its spectrum:

The stability of FEM simulation depends on the temporal and spatial resolution of the analysis. The stability limit for the integration time (time of travel) is *L*/*C_L_*. The parameters *L* and *C_L_* represent pipe length and bulk longitudinal wave velocity, respectively. The maximum frequency *f_max_* determines maximum time step Δ*t* and element size [48]:

(7) $$\Delta t=1/20f_{\max}$$

The element size is usually derived from the smallest wavelength (biggest frequency). A rigorous condition of 20 nodes per wavelength (*Le* = *λ_min_*/20) is recommended [48].

**Figure 5:**
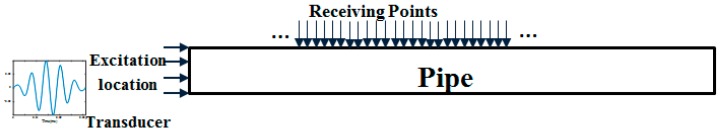
Collecting guided wave signals at every 1 mm along the pipe axis.

According to the dispersion curves depicted in Figure 1, the least dispersive region for L(0,2) mode starts roughly from 100 kHz to 240 kHz. In order to design an over-complete dictionary that includes this region, we swept the frequency from 100 kHz to 240 kHz with the step of 5 kHz. In each frequency step, the phase *θ* was kept constant equal to zero. Excitation of guided waves was carried out by applying a time-varying displacement according to Equation (3) on one end of the pipe. Guided wave signals were collected at every 1 mm from the excitation point (Figure 5). It must be noted that; in practice, a reflection of the signal from a defect will result in a phase shift. The reflected signal has the reverse phase of the direct one [49]. For considering the reversed-phase time-transient responses in our dictionary, instead of repeating the simulation with *θ* = *π*, to reduce computational cost we used Fast Fourier Transform (FFT). Doubling the size of our dictionary, it will include both direct signals with *θ* = 0 and reflection signals with *θ* = *π*.

## 4. Proposed SDMP Algorithm for Sparse Representation

The algorithm consists of two stages. In the first stage, a signal is approximated by MP. Using an appropriately designed, over-complete and redundant dictionary, results in a highly sparse representation. However, sparsity is not the only characteristic we seek in a signal representation for guided wave inspection. What is of the greater importance together with the sparsity is the physical interpretation of the resulting approximation. One of the major encountering issues in the ultrasonic inspection is the problem of overlapping different wave-packets in signals [50,51,52,53,54,55,56]. Addressing this problem, MP has been developed to more sophisticated and feasible versions [29,56]. For example, the approximation in each iteration can be improved by compromising between reducing the energy of the resulting residue *r*(*t*) and its “robust support” defined as:

(8) $${\left\|r_{n}\left(t\right)\right\|}_{q}=\sum_{i=1}^{N}{\left|r_{n}\left(t_{i}\right)\right|}^{q},\;0\;<<\;q\;<<\;1$$

which for *q* ≈ 0, gives the number of *t_i_*s with *r*(*t_i_*) ≠ 0 that coincide with the theoretical support [28]. In doing so, at iteration *k*, the correlation coefficients between the residue *r_k−_*_1_ and the dictionary *D* excluding its previously selected atoms are calculated. All atoms whose absolute correlation coefficients are above a predefined threshold *Th*_1_ are selected. Choosing an atom from this set ensures a significant reduction in $\|r{\left(t\right)\|}_{2}$. A chosen atom at each iteration must minimize “robust support” [56,57]:

(9) $$\arg\min{\left\|r_{n}(t)\right\|}_{q}=\arg\min\{\sum_{i=1}^{N}{\left|r_{n}\left(t_{i}\right)\right|}^{q}\}$$

While greatly improving conventional matching pursuit, the performance of this method degrades in situations of low SNR and highly overlapping echoes [52]. In our work, however, instead of correlation, we used the inner product. At iteration *k* unlike conventional matching pursuit, in which an atom that produces the maximum inner product with *r_n−_*_1_(*t*) is selected; initially, atoms whose inner product are above a threshold *Th*_1_ are chosen:

(10) $$Th_{1}=\alpha \arg\max\left|\langle r_{n}\left(t\right),a_{n+1}\rangle \right|;\;0.5\;<\;\alpha \;<\;1$$

in which *α* is a real number close to one and depends on a signal itself. After that, for every selected atom *r_n_*(*t*) is computed. The atom that generates the minimum norm according to Equation (5) is the final pick at iteration *k*.

**Figure 6:**
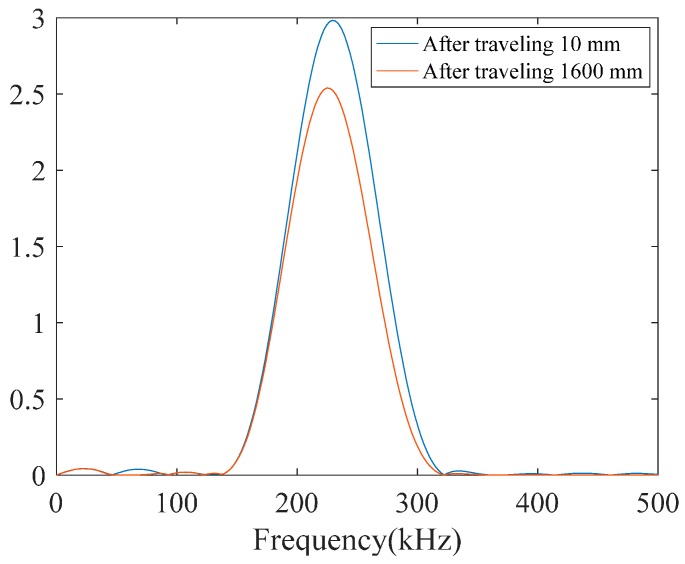
Frequency response of the L(0,2) mode tone-burst wave packet that emitted at a center frequency of 220 kHz and its bandwidth after traveling 10 mm and 1600 mm along the pipe.

Idealistically, when using a narrowband signal for excitation of guided waves, a narrowband response within the same frequency band is expected to be received. However, in practice, guided wave signals are contaminated by modal noise, mode reverberation, and other environmental factors so that in the frequency domain they are different than the excitation signal. Meanwhile, as a result of dispersion, the FFT of wave-packets changes after traveling some distance (Figure 6). Therefore, as mentioned earlier a good dictionary for matching pursuit must not only contain atoms with the same frequency characteristics as the excitation signal but also includes ones covering the proximity of the center frequency of the excitation wave-packet. After approximation of the signal with the matching pursuit, in the second stage, we aim to retain only the atoms that have frequency consistency with the center frequency of the excitation signal. If such atoms are selected and other atoms discarded from the matching pursuit approximation, as a result, the representing signal will only contain atoms that carry meaningful information about the structure.

In the second stage, we built another dictionary (*D’*) that contains atoms in the frequency domain. The center frequencies for these atoms (which are assumed to be the excitation signals) starts from 100 kHz to 240 kHz with the step of 10 kHz. Therefore, this dictionary consists of 15 tone-burst signals in the frequency domain. It must be noted that this interval can be changed according to the data obtained from experiments. For the next step, since guided wave signals are idealistically supposed to have the same frequency components as the excitation signal, the atoms whose frequencies are consistent with the center frequency *f_c_* of the excitation signal are selected. Meanwhile, as dispersion may slightly shift the center frequencies of propagating wave packets, the nearby frequencies in the dictionary must be taken into account as well as *f_c_* itself. Therefore, in the second stage of our algorithm, we discard the atoms whose correlation in the frequency domain with the FFT of the excitation signal is less than a predefined threshold. The value for this threshold depends upon the nature of our signal. It must be noted that atoms whose frequencies are very different than the center frequency of the excitation signal cannot be related to ideal guided wave signal representing the features of the structure.

For the best and sparse representation of a guided wave signal, initially, the signal is approximated by the redundant and over-complete dispersive dictionary described in stage one. In the second stage, as some atoms with the frequency inconsistency are discarded, the signal is represented with an even smaller number of atoms meaning sparser with clear physical meanings.

The proposed algorithm works for approximating a guided wave signal *x*(*t*) with *n* ≥ 1 atoms is as follows:

Phase I.
  (1) Initialize the iteration counter *n* = 0 and the residue *r_n_*(*t*) = *x*(*t*).
  (2) Calculate the inner product between the current residue *r_n_*(*t*) and all atoms in *D* excluding ones that were already selected in the previous iterations.
  (3) Select all atoms whose inner product with *r_n_*(*t*) are above a specific threshold *Th*_1_ value:
    (11) $$A=\left\{a\left(t\right)|\left|\langle a(t),r_{n}(t)\rangle \right|\geq Th_{1}\right\}$$
    (12) $$Th_{1}=\alpha \arg\max\left|\langle r_{n}\left(t\right),a_{n+1}\rangle \right|;\;0.5\;<\;\alpha \;<\;1$$
  (4) Calculate the next residual signal *r_n+_*_1_(*t*) for each atom selected in the previous Step (3):
    (13) $$r_{n+1}\left(t\right)=r_{n}\left(t\right)-\langle r_{n}\left(t\right),a\left(t\right)\rangle a\left(t\right);a\left(t\right)\in A$$
  (5) Choose the atom that minimizes the robust support and put it in *Â*:
    (14) $$\hat{A}=\left\{a_{n+1}\left(t\right)|\arg\min{\left\|r_{n+1}\left(t\right)\right\|}_{q}\right\}$$
    (15) $$\arg\min{\left\|r_{n+1}\left(t\right)\right\|}_{q}=\arg\min{\left\|r_{n}\left(t\right)-\langle r_{n}\left(t\right),a_{n+1}\left(t\right)\rangle a_{n+1}\left(t\right)\right\|}_{q};a\left(t\right)\in A;0<<q<<1$$
  (6) Update the residue based on the chosen atom.
  (7) If the residue energy ${\left\|r_{n+1}\left(t\right)\right\|}_{2}<\varepsilon$ then stop, otherwise *n* = *n* + 1 and jump to Step (2).
Phase II. Increasing Sparsity.
  (8) Calculate the FFT of the atoms in *Â*:
    (16) $$B=\left\{a\left(f\right)|a\left(f\right)=FFT\left(a\left(t\right)\right)\right\};a\left(t\right)\in \hat{A}$$
  (9) Calculate the absolute cross-correlation *c* between the atoms in *B* and the atom in *D’* representing excitation signal at the center frequency (*b_fc_*).
  (10) Discard the atoms in *Â* whose absolute cross-correlation in the frequency domain with *b_fc_* is smaller than a predefined threshold *Th*_2_.

## 5. Demonstration of the SDMP Capabilities

Validation of the proposed SDMP algorithm for the sparse representation of guided wave signals takes place in two parts. In the first part, the detectability of a defect with regard to SNR is investigated and compared with the conventional matching pursuit. This is demonstrated by simulation data from a steel pipe. In the second part, the capability of this method in mode separation is verified by simulation data and experimental results.

### 5.1. FEM Simulation

**Figure 7:**
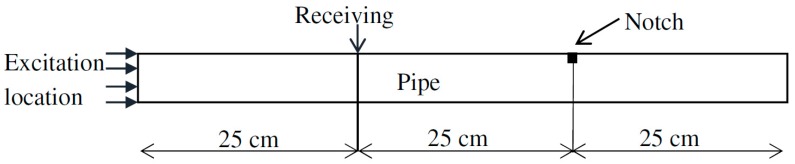
Schematic diagram of the FEM model representing a pipe with a small notch (crack).

When it comes to the application of guided waves in practical cases, environmental and coherent noise must be considered for accurate defect characterization. As in real cases, a defect indication in a signal may be covered by such noise; the tolerance of the proposed method SDMP in an adverse environment is examined. Figure 7 demonstrates the schematic diagram of FEM model used for simulating a steel pipe with a notch. The 75 cm length pipe with 33 mm diameter and 4 mm wall thickness had a notch located on 50 cm from the excitation point. The depth of the notch was chosen 10% of the pipe wall thickness to best demonstrate the capability of our algorithm in approximating the reflection of small defects.

**Figure 8:**
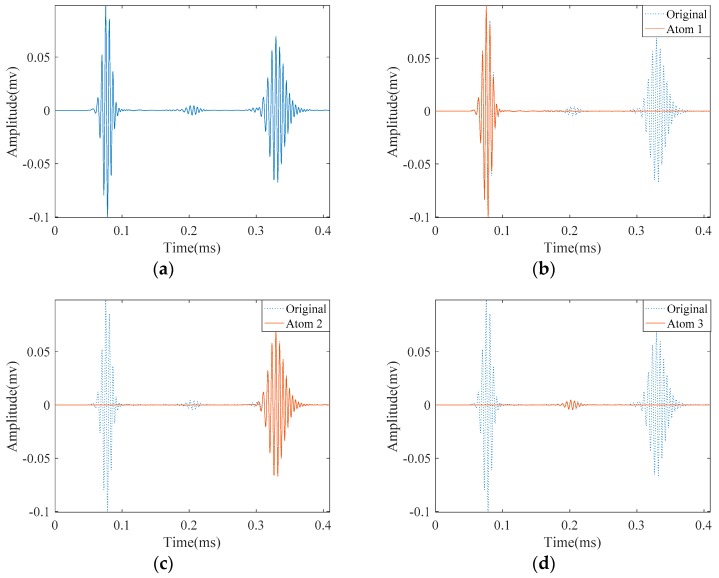
Results obtained by applying SDMP to the simulation signal in a pipe with a small crack: (**a**) the original signal of the propagating guided wave; (**b**) approximated atom 1—the direct signal; (**c**) approximated atom 2—the reflection from the pipe end; and (**d**) approximated atom 3—the reflection from the crack.

Excitation of longitudinal L(0,2) mode was carried out by applying a prescribed displacement in the form of Equation (3) with a center frequency of 180 kHz on one end of the pipe. In the pitch-catch mode for such pipe three wave-packets are visible in the signal as shown in Figure 8a. They represent a direct signal passing from the receiving point, the reflection from the notch and from the right end of the pipe, respectively. A good approximation of this signal must decompose it with regard to theses wave-packets. For the SDMP algorithm, we chose the robust support *q* = 0.1, *α* = 0.95, and *Th*_2_ = 0.8 which provide good results. These parameters are tabulated in Table 2 together with the parameters of the conventional MP.

The approximation of this noise-free signal with SDMP is depicted in Figure 8. The original signal is visible with dashed lines in the background; while approximated atoms are illustrated with solid lines. As the direct signal passing by the receiving point has the highest amplitude and energy, it is approximated as the first atom. Similarly, the reflections from the pipe end and the defect are approximated as the second and the third. Comparing these atoms with the original signal in the background with the dashed lines, a good consistency can be observed. Depending on the value of *ε*, if the iteration continues, more atoms will be approximated to reduce the *ε*. However, after successfully approximating the signal with three atoms, the extra approximation will not bear meaningful information.

If the signal *x*(*t*) in Figure 8a is corrupted by a random Gaussian noise *e*(*t*), the noisy signal $\hat{x}\left(t\right)$ can be written as:

(17) $$\hat{x}\left(t\right)=x\left(t\right)+e\left(t\right)$$

where the signal-to-noise ratio (SNR) is defined as

(18) $$\mathrm{SNR}=10\log_{10}\frac{\sum_{i=1}^{N}{\left|x\left(t_{i}\right)\right|}^{2}}{\sum_{i=1}^{N}{\left|e\left(t_{i}\right)\right|}^{2}}\left(\mathrm{dB}\right)$$

**Figure 9:**
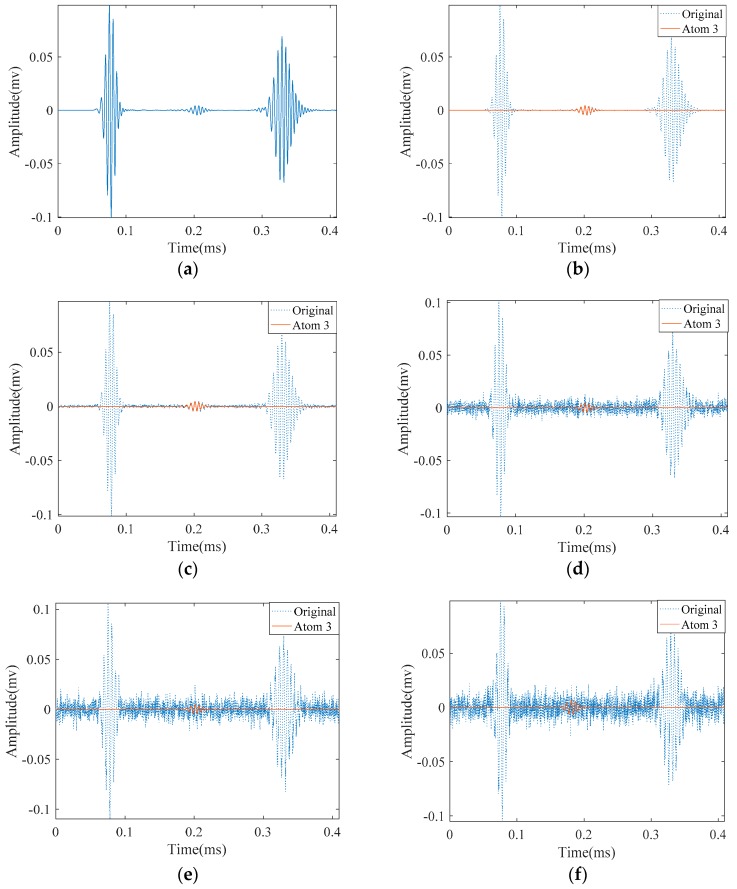
Detection of the small crack in the presence of noise with different signal to noise ratios (SNRs) with Sparse Representation with Dispersion Based Matching Pursuit (SDMP): (**a**) the original signal; (**b**) the approximation of crack reflected signal without noise; and with added noise that has: (**c**) SNR = 23.8 dB; (**d**) SNR = 9.8 dB; (**e**) SNR = 6.8 dB; and (**f**) SNR = 5.8 dB.

**Figure 10:**
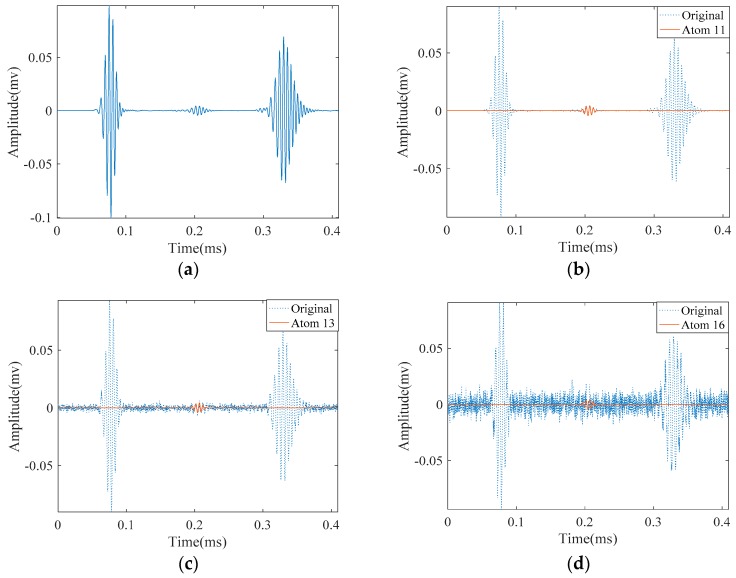
Detection of small defect in the presence of noise with different SNRs with MP: (**a**) the original signal; (**b**) approximation of crack reflected signal without noise; and (**c**) with added noise SNR = 23.8 dB; and (**d**) with added noise SNR = 9.8 dB.

With different values of SNR, we investigate the capability of our proposed method SDMP to approximate the defect signal in Figure 8. Idealistically when we have a pure guided wave signal without noise, we can approximate the defect signal at the third iteration of SDMP as the third atom (Figure 8). Figure 9 demonstrates the approximation of the signal reflected by the defect in the presence of different noise levels. For a high SNR value such as 23.8 dB, in which the reflection of the defect is visible, the defect signal is robustly approximated as the third atom. For lower values of SNRs, in which the defect signal is hidden, up until SNR = 6.8 dB, the defect signal is exposed despite being hidden in the original signal. When the noise level goes higher; for lower SNR values starting from SNR = 5.8 dB, SDMP cannot locate the defect and misrepresent the defect location. For the comparison purpose, we later applied MP with a Gaussian dictionary on the same signals in Figure 9. The results are depicted in Figure 10. As can be seen, MP is very sensitive to the noise and cannot approximate the defect signal as the third atom. If we let the iteration continues in MP, it may finally locate the defect indication hidden in the noise. This will cost at the expense sparsity and in fact, it will sacrifice it for the defect detection purpose. As the noise level goes higher (lower SNR), the sparsity deteriorates up to a point in which the MP cannot locate the defect at all.

**Figure 11:**
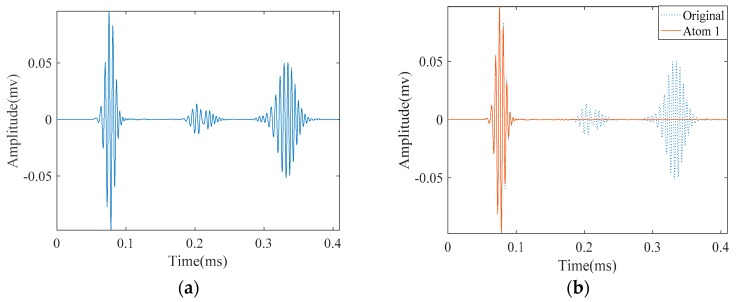

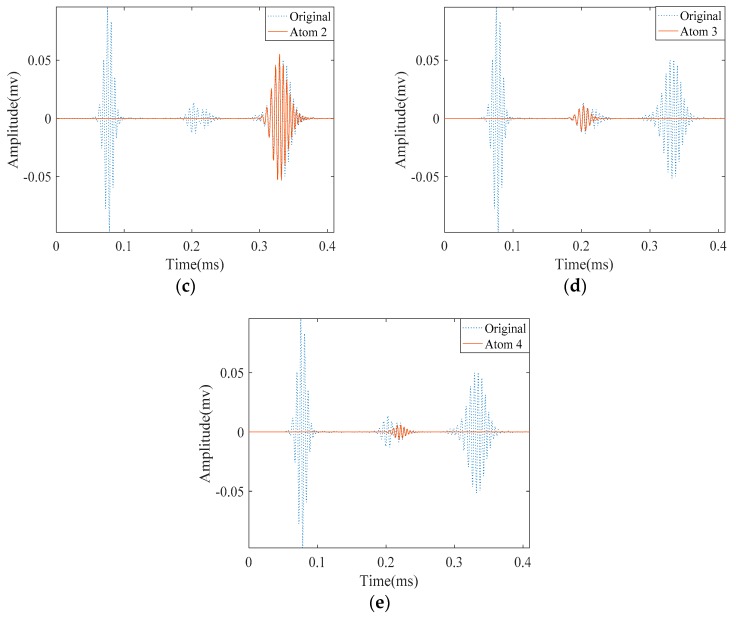
Results obtained by applying SDMP to the simulation signal in a pipe with 27 mm length notch: (**a**) the original signal; (**b**) approximated atom 1—the direct signal; (**c**) approximated atom 2—the reflection from the pipe end; (**d**) approximated atom 3—the reflection from the first edge of the notch; and (**e**) approximated atom 4—the reflection from the second edge of the notch.

**Figure 12:**
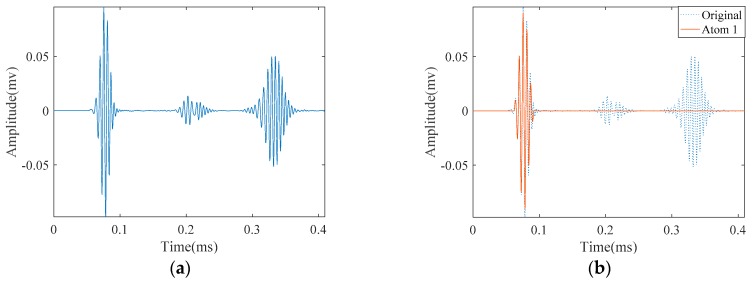

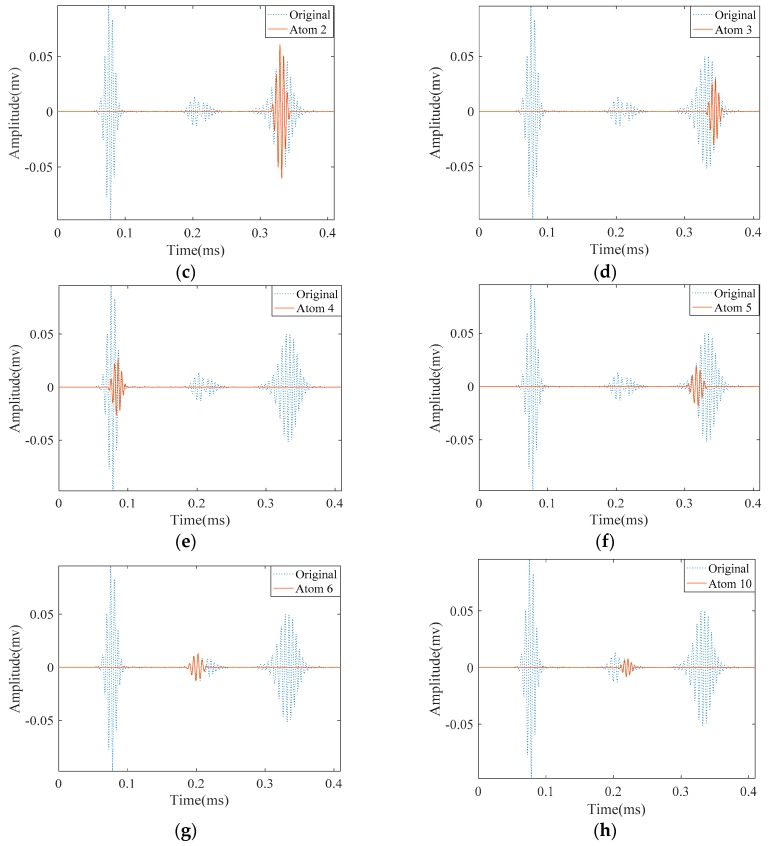
Results obtained by applying MP to the simulation signal in a pipe with the 27 mm length notch: (**a**) the original signal; (**b**) approximated atom 1—the direct signal; (**c**) approximated atom 2—the reflection from the pipe end; (**d**) approximated atom 3—the reflection from the pipe end; (**e**) approximated atom 4—the direct signal; (**f**) approximated atom 5—the reflection from the pipe end; (**g**) approximated atom 6—the reflection from the first edge of notch; and (**h**) approximated atom 10—the reflection from the second edge of notch.

In order to investigate the robustness of the SDMP in separating overlapped wave-packets, the simulated notch was extended along the pipe axis. The newly simulated notch had 27 mm length. The temporal waveform obtained from this structure is depicted in Figure 11. As can be seen, unlike the crack in Figure 8a, the reflection from the defect consists of two overlapped wave-packets. They stand for the two edges of the notch. Our primary objective in here is to separate these overlapped components and represent the signal with the maximum sparsity. Without changing the parameters tabulated in Table 2, we applied SDMP to this signal. As expected, because of having higher energy, the direct signal and the reflection from the pipe end were approximated as the first and second atoms, respectively. The reflections from the two edges of the notch were approximated as the third and fourth atoms. This response is the sparsest response that represents the signal with overlapped components. If the same signal is approximated by MP with a Gaussian dictionary and the parameters in Table 2, the response will not be as sparse as the one with SDMP. In Figure 12, we can see the decomposition and approximation of the same signal by MP with a Gaussian dictionary. In the first and second iteration of the MP algorithm, direct signal and the reflection from the pipe end were successfully approximated. Nonetheless, as the atoms in the dictionary do not match well with the existing wave-packets in the signal, in later steps, the algorithm still tries to compensate the poor representation by selecting more atoms in the dictionary around the same location. If the iteration continues, the first edge will appear as the sixth atom. For the same reason mentioned for the direct and reflection from the end wave packets, the reflection from the second edge of the notch will not be visible until the tenth iteration. Although MP finally locates the two edges of the defect signal, the final result is not sparse. Being not sparse, MP with the Gaussian dictionary could complicate signal representation, by falsely selecting atoms in its dictionary that do not carry meaningful information.

### 5.2. Experimental Results

**Figure 13:**
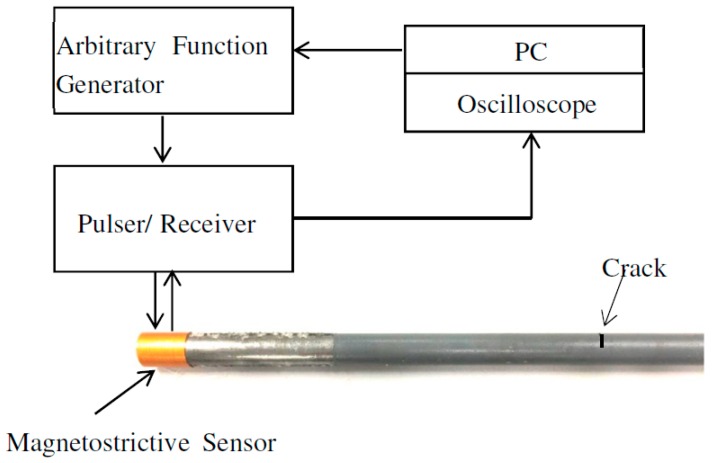
The schematic diagram of the experimental setup.

In practice, the success of SDMP depends on its performance in representing real experimental data. In order to demonstrate the potential capabilities of the proposed method, the experiment was conducted on an 80 cm long steel pipe. It was standard pipe delivering natural gas to residential buildings with the diameter of 33 mm and 4 mm wall thickness. For corrosion protection, such standard pipes have thin multilayer cover consists of zinc epoxy and acrylic. A crack representing a defect was artificially made on the pipe with a saw. In our experiment, we used magnetostrictive sensor (MsS) for exciting and receiving L(0,2) mode in pulse-echo mode. The MsS sensor consists of flexible printed coil (FPC) [58] and a smart material which is a highly ferromagnetic patch [59]. The role of the patch is to enhance magnetostriction effect and consequently SNR. In this patch, guided waves can be generated and detected using the Joule and Villari magnetostrictive effects which refer to a physical dimensional change due to an application of magnetic field or, inversely, a magnetic flux change due to an application of mechanical strain [60]. For the excitation of L(0,2) mode at the selected frequency five-cycle tone-burst signal modulated by Hamming window according to Equation (3) was delivered through an arbitrary signal generator and generated by an RITEC 4000 pulser and receiver (RITEC Inc., Warwick, RI, USA). Figure 13 shows the schematic diagram of the experimental setup employed for guided-wave-based damage detection applications.

**Figure 14:**
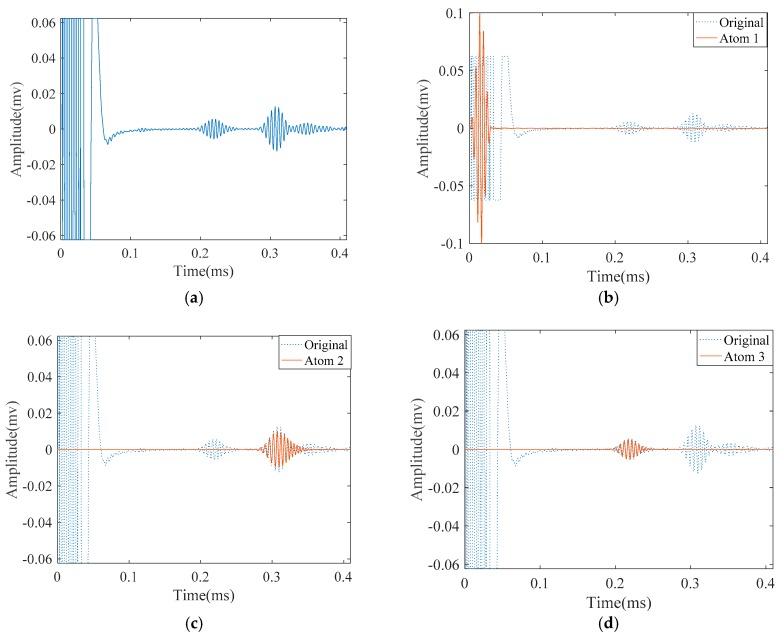
The experimentally measured signal in a cracked pipe and its approximation by SDMP: (**a**) the original measured signal; (**b**) approximated atom 1—the incident signal; (**c**) approximated atom 2—the reflection from the pipe end; and (**d**) approximated atom 3—the reflection from the crack.

Figure 14a illustrates the measured signal from the abovementioned experimental setup. Unlike the signals obtained from FEM simulation, the signal is contaminated with some unwanted components. According to the parameters in Table 2, we tried to approximate this signal with SDMP. Despite having a contaminated signal, consistent with the simulation result, SDMP represents the signal with maximum sparsity. The first wave packet to be approximated as a first atom is the incident signal. In the position of the incident signal, there is electrical leakage from the equipment. However, as SDMP checks the frequency domain as well as the time domain for the selected atoms, only one atom is assigned to that location. The second and third atoms are representing the reflection from the end and the defect, respectively.

**Figure 15:**
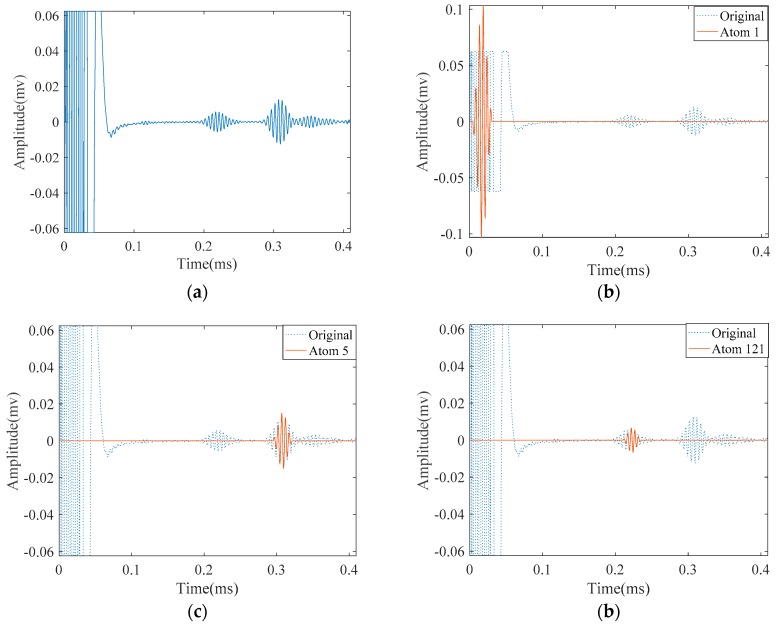
The experimentally measured signal in a cracked pipe and its approximation by MP: (**a**) the original measured signal; (**b**) approximated atom 1—the incident signal; (**c**) approximated atom 5—the reflection from the pipe end; and (**d**) approximated atom 121—the reflection from the crack.

Meanwhile, we applied MP with a Gaussian dictionary to the same signal. The results are demonstrated in Figure 15. Except for the first approximated atom, the results are not consistent with results from SDMP. The reflection from the pipe end appears as the third atom and the reflection from the defect does not emerge until the 121st atom. This means that MP with Gaussian dictionary needs at least 121 iterations to separate the defect indication in this signal. This happens as a result of the poor approximation at each iteration of MP. MP tries to compensate this poor approximation by adding more and more atoms in its next iterations which consequently increases the number of selected atoms for representing the signal. Apart from being computationally expensive, as was already mentioned for the FEM simulation signal, this representation can mislead signal interpretation by approximating atoms that do not carry physically meaningful information structure.

**Figure 16:**
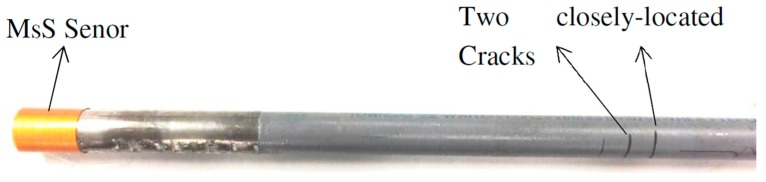
The real gas pipe with two closely located cracks.

In the second of our experiment, in order to further verify the capability of SDMP in separating overlapped modes, we conducted an experiment on the same pipe with some alteration. We added another crack on the same pipe that had a 22 mm distance in the axial direction from the initial crack. The two cracks together with MsS Sensor on the pipe are shown in Figure 16.

**Figure 17:**
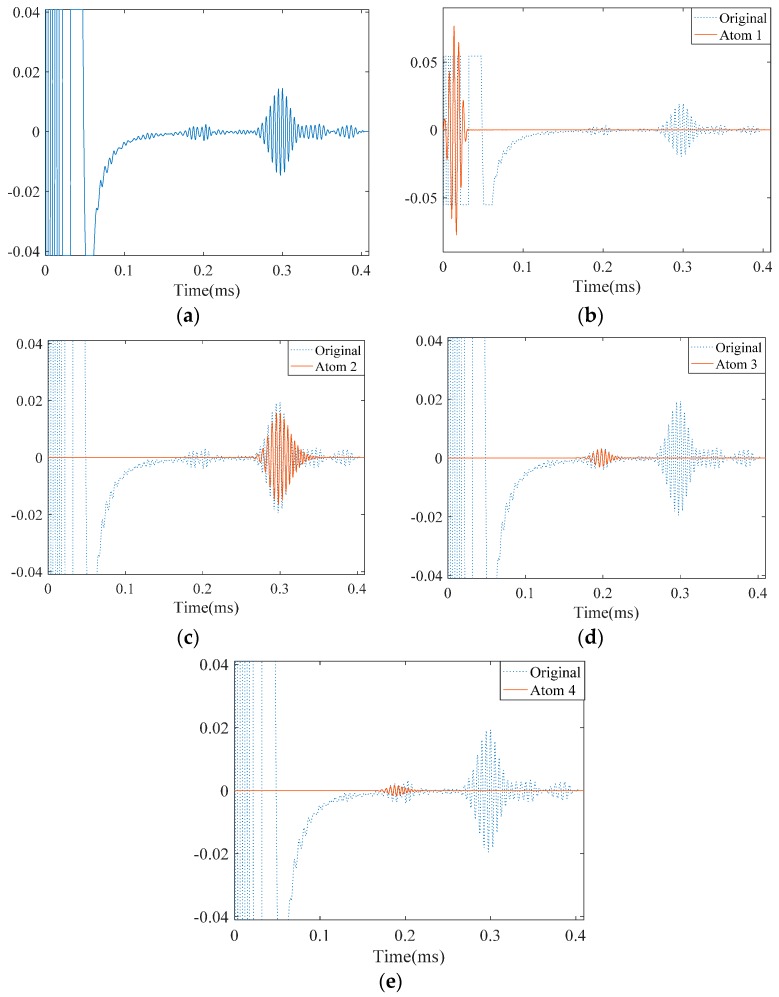
The experimentally measured signal in a pipe with two cracks and its approximation by SDMP: (**a**) the originally measured signal; (**b**) approximated atom 1—the incident signal; (**c**) approximated atom 2—the reflection from the pipe end; (**d**) approximated atom 3—the reflection from the first crack; and (**e**) approximated atom 4—the reflection from the second crack.

Since the distance is close, the two wave packets reflecting from them are overlapped (Figure 17a). We applied SDMP to this signal to see if they can be separated. Similar to the case with one crack, the first approximated atom refers to the incident signal as it has the highest energy at the location of the sensor (Figure 17b). For the same reason, the second atom indicates the reflection from the pipe end. As can be seen in Figure 17, after approximating first and second atoms representing the incident signal and reflection from the end, respectively, the reflections from the two cracks are represented as the third and fourth atoms.

**Figure 18:**
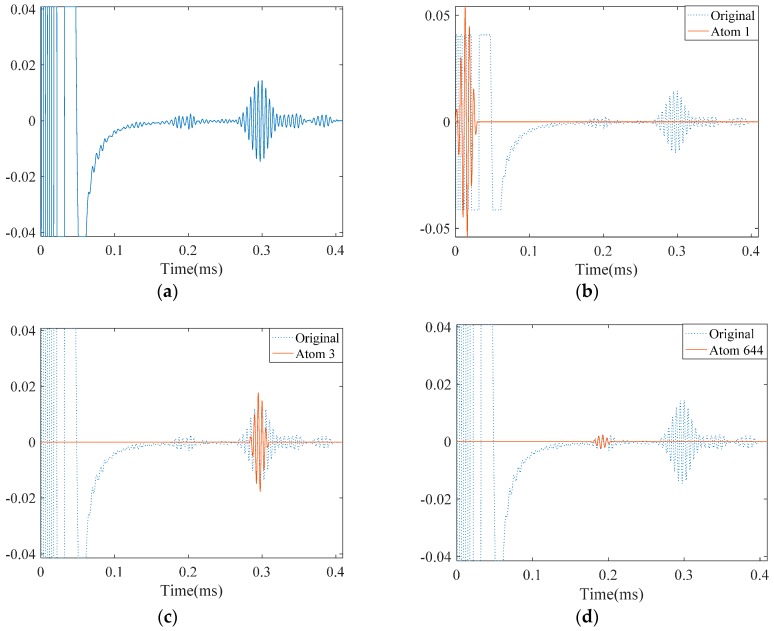
The experimentally measured signal in a pipe with two cracks and its approximation by MP: (**a**) the originally measured signal; (**b**) approximated atom 1—the incident signal; (**c**) approximated atom 2—the reflection from the pipe end; (**d**) approximated atom 3—the reflection from the first crack; and (**e**) approximated atom 644—the reflection from the second crack.

For the purpose of comparison, we applied MP with a Gaussian dictionary to the same signal. As can be seen in Figure 18, the first atom, similarly other study cases, approximates the incident signal at the location of the sensor. The reflection from the pipe end takes place as the third atom which is similar to the case of the pipe with one crack. However, as these two approximations were poor, MP tries to improve the representation by selecting more irrelevant atoms that do not have physical meanings. We let the MP continue its iteration and, as a result, the first defect signal appeared as the 664th atom. The reflection from the second crack could have emerged if we had further pursued the iterations in MP. However, this result clearly demonstrates how the SDMP enhances the performance of the guided wave signal representation.

## 6. Conclusions

This paper presents a new approach for approximation and interpretation of guided wave signals for non-destructive testing of pipes. An advanced signal processing algorithm called SDMP was designed based on dispersion characteristic of guided waves. An over-complete dictionary is constructed taking dispersion into consideration. FEM simulation was used to design such dictionary that precisely predicts the waveform propagating along the structure. With the SDMP, which is a two-stage improved matching pursuit (MP), a noisy signal is efficiently decomposed and hidden defect signal can be revealed. In the first stage of the algorithm, main features of a signal are extracted. In the second stage, the atoms that are selected to approximate the signal are evaluated to see if they have frequency consistency with the excitation signal. The inconsistent atoms in the second stage are then discarded to provide the sparse representation of the signal with actual physical meaning. The applicability of SDMP was verified by FEM simulation and experimental data. The SDMP successfully detected indications of the defects buried in the noise. Thus, it can be concluded that it has a good performance when SNR is low. Meanwhile, using this algorithm, the overlapped wave-packets that frequently occur in guided wave signals were successfully separated. In comparison with conventional matching pursuit, the SDMP with dispersive dictionary has greatly enhanced the performance of matching pursuit and guarantees the maximum sparsity.

Although the presented SDMP for signal interpretation addresses the inspection of steel pipes, it can be applied to any plate-like structure made from different materials such as plastic pipes, aluminum and composite plates, steel strands and rails. The only difference is to design a new dictionary based on FEM simulation for a new material and geometry. As any irregularity in their geometry will reflect back part of the guided wave energy, cracks, corrosion, joints, etc. can be identified. Meanwhile, by designing such dictionaries in systems consisting of different structures, the whole system can be inspected and monitored for structural integrity.

## Figures and Tables

**Table 1:** The parameters used in the Finite Element Method (FEM) simulations.

Density (*ρ*)	Young’s Modulus (E)	Poisson’s Ratio (λ)
7800 kg/m^3^	200 GPa	0.33

**Table 2:** Parameters that were used for approximation of signals obtained from FEM.

Method	*q*	α	*Th*_2_
SDMP	0.1	0.95	0.8
MP	0.1	1	-
